# Evaluation of the Loop-Mediated Isothermal Amplification Assay (LAMP) Eazyplex^®^ *Pneumocystis jirovecii*

**DOI:** 10.3390/jof11040300

**Published:** 2025-04-10

**Authors:** Ulrike Scharmann, Lisa Kirchhoff, Jan Buer, Franziska Schuler, Annerose Serr, Susann Rößler, Jürgen Held, Tobias Szumlanski, Joerg Steinmann, Peter-Michael Rath

**Affiliations:** 1 Institute of Medical Microbiology, University Hospital Essen, University of Duisburg-Essen, 45147 Essen, Germany; lisa.kirchhoff@uk-essen.de (L.K.); jan.buer@uk-essen.de (J.B.); joerg.steinmann@klinikum-nuernberg.de (J.S.); peter-michael.rath@uk-essen.de (P.-M.R.); 2 Institute of Medical Microbiology, University Hospital Münster, 48149 Münster, Germany; franziska.schuler@ukmuenster.de; 3 Institute of Medical Microbiology and Hygiene, Medical Center-University of Freiburg, Faculty of Medicine, University of Freiburg, 79104 Freiburg, Germany; annerose.serr@uniklinik-freiburg.de; 4 Institute of Medical Microbiology and Virology, University Hospital Carl Gustav Carus, TU Dresden, 01307 Dresden, Germany; susann.roessler@tu-dresden.de; 5 Institute of Microbiology—Clinical Microbiology, Immunology and Hygiene, University Hospital Erlangen, 91054 Erlangen, Germany; juergen.held@uk-erlangen.de; 6 Institute of Clinical Microbiology, Infectious Diseases and Infection Control, Paracelsus Medical University, Nuremberg General Hospital, 90419 Nuremberg, Germany; tobias.szumlanski@klinikum-nuernberg.de

**Keywords:** LAMP, qPCR, *Pneumocystis jirovecii*, molecular diagnostic, fungal infection, pulmonary infection

## Abstract

A commercially available loop-mediated isothermal amplification assay (LAMP) for the detection of *Pneumocystis jirovecii* (*P. jirovecii*) has been evaluated for the diagnosis of *Pneumocystis* pneumonia (PcP) in critically ill patients. Altogether, 109 lower respiratory tract specimens from 95 patients with a positive *P. jirovecii* test in routine diagnostics were collected from five distinct university hospitals in Germany. All samples were tested with a qPCR and eazyplex^®^ LAMP assay. qPCR was set as the gold standard and was evaluated beforehand with samples from 100 patients categorized to have proven, probable, and possible PcP according to the EORTC/MSGERC guidelines. The sensitivity, specificity, and positive and negative predictive value (PPV and NPV) of the LAMP were assessed. Sensitivity was 68%, specificity was 86%, and PPV and NPV were 99% and 16%, respectively. All patients with proven PcP were positive in the LAMP. There was a weak correlation between the time to positivity and the fungal load (squared Pearson correlation coefficient (r^2^) = 0.5653). A positive result in the LAMP indicates a PcP. Because of the low sensitivity, negative results do not rule out an infection and should be clarified with further molecular methods. The LAMP should be used in patients in whom a PcP is expected, not for screening only.

## 1. Introduction

In patients with HIV/AIDS, *Pneumocystis* pneumonia (PcP) caused by *Pneumocystis jirovecii* (*P. jirovecii*) is an important cause of opportunistic fungal infections [1]. In addition, the incidence of PcP in non-HIV/AIDS patients is rising due to the increased use of immunosuppressive agents; thus, a reliable diagnosis of PcP is becoming more important [2]. The diagnosis is crucial due to various criteria, which are non-fungal-specific. The infectious diseases group of the European Organization for Research and Treatment of Cancer and the Mycoses Study Group (EORTC/MSG) defined three classes for the IPA (proven, probable, and possible) depending on which consensus criteria are fulfilled [3]. These criteria include host factors of the patients (e.g., previous illnesses or immunosuppressive therapy), clinical features (radiological signs, abnormalities in typical anatomical sides), and mycological evidence (culture or microscopical detection of fungal structures in respiratory material and detection of biomarkers, like galactomannan (GM) antigen or (1→3)-β-D-glucan (BDG) and DNA detection) [3]. However, once PcP is diagnosed, first-line antibiotic therapy with high-dose co-trimoxazole should be started immediately [4]. Since therapy with high-dose co-trimoxazole can have severe side effects for critically ill patients, it should only be applied if necessary. The guidelines suggest an algorithm for diagnosing *P. jirovecii* in respiratory samples from patients with hematological malignancies and stem cell transplant recipients [5]. For diagnosis, microscopic immunofluorescence assays (IFAs) and/or the molecular detection of *P. jirovecii* is recommended, using bronchoalveolar lavage (BAL) fluid. Quantitative real-time PCR (qPCR) is carried out in some laboratories [5,6]. It is often challenging to distinguish between colonization and infection with *P. jirovecii* if the qPCR is positive. The cutoff for qPCR differs between laboratories, depending on the used assay and the patient cohort [6]. However, due to the high costs and often low number of requirements, this test is not feasible for every laboratory. As an alternative for qPCR, the loop-mediated isothermal amplification assay (LAMP) eazyplex^®^ *Pneumocystis jirovecii* (AmplexDiagnostics GmbH, Gars am Inn, Germany) has been commercially available since 2018. The LAMP is based on an isothermal amplification of DNA even when a low number of copies are present in the sample [7] and is based on targeting the mitochondrial gene cytochrome c oxidase subunit 2 (cox2). The advantage of the LAMP assay is that no DNA extraction is necessary. Only a specialized heater and reading block are necessary for analysis. The result appears after 25 min. The reagents of the assay are lyophilized, ready to use, and can be stored at room temperature.

Here, we evaluated the performance of the LAMP eazyplex^®^ *Pneumocystis jirovecii* on its suitability to detect *P. jirovecii* in human respiratory samples from five university hospitals in Germany.

## 2. Materials and Methods

### 2.1. Patients and Specimens

In total, 109 respiratory samples from 95 patients, from five university hospitals in Germany, that each tested positive for *P. jirovecii* in routine diagnostics (via microscopy and/or molecular detection) were investigated. Classification of the patient cohort was performed according to the EORTC/MSGERC criteria [3,8]. Patients with missing clinical data were defined as not classifiable. Invasive fungal infections are categorized to be proven, probable, and possible depending on host factors, clinical features, and mycological evidence [3,8]. Respiratory samples included bronchoalveolar lavage (BAL, *n* = 105), bronchial secretions (BS, *n* = 1), and induced sputum samples (*n* = 3).

All respiratory samples were investigated via qPCR at the Institute of Medical Microbiology of the University Hospital in Essen, an ECMM Excellence Center with diamond status. The LAMP assays were also performed here, except for the Erlangen samples, for which LAMP assays were performed at the Institute of Microbiology—Clinical Microbiology, Immunology and Hygiene, University Hospital Erlangen. The sampling period for the respiratory material was from 1 September 2020 to 1 September 2021. Samples were stored (a maximum of three days) at −20 °C until molecular testing was performed.

All clinical samples used in the present study were analyzed after conventional microbiological diagnostic tests had been performed. This study did not result in additional constraints for the patients. All analyses were carried out in accordance with the established guidelines. This study was approved by the local ethics committee (Ethics Committee of the Faculty of Medicine Essen, University of Duisburg-Essen, Essen, Germany; reference number 20-9399-BO, 6 August 2020).

### 2.2. PCR

PCR assays, including the qPCR RealStar *Pneumocystis jirovecii* PCR Kit 1.0 (altona Diagnostics GmbH, Hamburg, Germany) and the eazyplex^®^ *Pneumocystis jirovecii* LAMP assay (AmplexDiagnostics GmbH, Gars-Bahnhof, Germany), were performed according to the manufacturer’s guidelines, as previously described [9]. The qPCR targets the *multicopy mitochondrial large-subunit rRNA* (*mtLSU*) gene, and the LAMP is based on the *mitochondrial gene cytochrome c oxidase subunit 2* (*cox2*).

In brief, for qPCR, DNA extraction was performed in a Maxwell16 instrument (Promega GmbH, Walldorf, Germany) with the Maxwell16 Tissue LEV Total RNA Purification Kit (Promega Corporation, Madison, WI, USA). The qPCR was performed in a RotorGeneQ thermocycler (Qiagen, Hilden, Germany). The fungal load in copies/mL in the eluate was determined, with a formula provided by the manufacturer regarding the internal standards and the cycle threshold (Ct) of the sample:

$$Fungal\;load\left(Sample\right)\left[copies/mL\right]=\frac{Volume\left(Eluate\right)\left[µL\right]\cdot Fungal\;load\left(Eluate\right)\left[copies/µL\right]}{Sample\;Input\;[mL]}$$

For the LAMP, 25 µL of sample was mixed up with 500 µL of buffer and incubated for 3 min at 99 °C and then added to lyophilized reagents. The LAMP was performed in a Genie^®^ II Mk 2 device (AmplexDiagnostics GmbH). The test run was ready after a maximum of 25 min. Interpretation of the results was automatically performed by the eazyReport^TM^ software (version 2.34.3) of the Genie^®^ II Mk 2 device. The results were reported as positive, negative, or invalid, along with a display of time to positivity (TTP) in minutes (min).

As the positive control, the external quality control assessment schemes (INSTAND, Düsseldorf, Germany) were used. Negative controls were implemented by using induced sputa from healthy individuals (*n* = 3).

To ensure the lack of influence of storage conditions on assay performance, three samples that tested positive in routine diagnostics were assessed: LAMP assays were performed before storing these samples at −20 °C for two years and then thawing them for re-evaluation. After this procedure, the samples were retested on three consecutive days, during which they were stored at room temperature.

### 2.3. Evaluation of qPCR as Gold Standard

**Figure 1:**
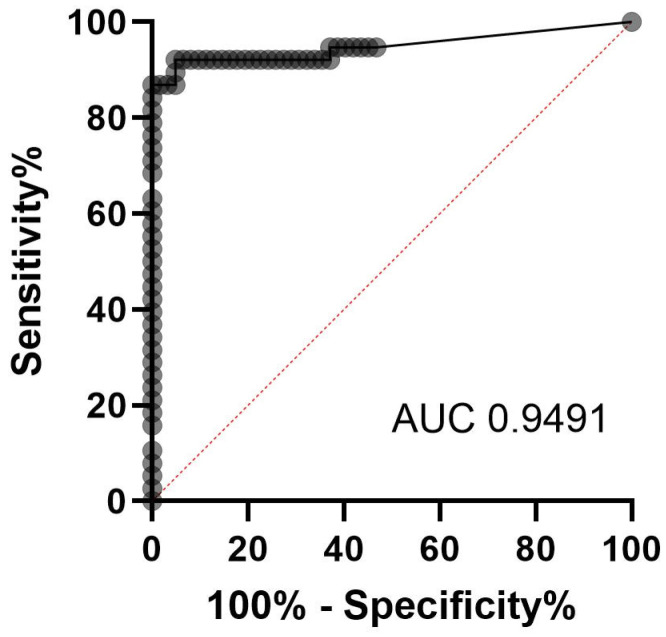
ROC analysis revealed an AUC of 0.9491 and a Ct higher than 29.29 to differentiate between a colonization and an infection with *P. jirovecii*.

Receiver operating characteristic (ROC) analysis was performed to define a Ct for the qPCR as a threshold based on clinical features, enabling differentiation between a positive and negative result. For this purpose, 100 respiratory specimens of 100 different patients, which were sent to the laboratory for routine *P. jirovecii* diagnostics between the years 2020 and 2023, were categorized to have proven, probable, possible, or no PcP, in accordance with the revised EORTC/MSGERC guidelines [3,8]. These patients were different from those used to evaluate the LAMP assay. ROC curve analysis (Figure 1) was performed using GraphPad Prism (version 8.0.0 for Windows, GraphPad Software, San Diego, CA, USA, www.graphpad.com). Ct values from patients categorized to have a proven and probable PcP were summarized as positive and set against Ct values of patients, categorized as possible and not expected PcP, which were summarized in true negative results. The Ct value with the highest sensitivity and specificity was set as the threshold to differentiate between a colonization and an infection with *P. jirovecii*.

### 2.4. Statistical Analysis

For quantification purposes, the TTP of the LAMP was plotted against the Ct in the qPCR. The threshold of the qPCR was set as the gold standard. Negative and positive predictive values (NPVs and PPVs), sensitivity, and specificity were calculated according to true and false negative as well as positive results in the LAMP in accordance with the qPCR. A true positive was defined as the Ct of qPCR being <29.29 and the LAMP simultaneously indicating a positive result. Linear correlation was calculated using the squared Pearson correlation coefficient (r^2^) (GraphPad Prism). *p* values of <0.05 were considered significant. For this, negative LAMP results were set as TTP 40 min in accordance with a negative result in the qPCR. Since there is no numerical value for a negative LAMP result, we have defined the negative results as TTP 40 min. This was carried out in relation to the Ct value of the qPCR, which is also considered negative at Ct 40.

## 3. Results

### 3.1. Evaluation of qPCR as Gold Standard

The categorization of the patients only included in the ROC analysis consisted of 31 probable, 7 possible, and 62 negative patients for PcP. The AUC was 0.9491 (0.8925 to 1.000 95% confidence interval, *p* value < 0.0001). The ROC analysis is depicted in Figure 1. The optimal Ct cutoff of the qPCR with the highest sensitivity as a leading marker combined with the associated highest sensitivity was 29.29. A Ct of 29.29 showed a sensitivity of 86.84% (95% CI 72.67–94.25%) and a specificity of 100% (95% CI 94.17–100%) and differentiates between a colonization and an infection with *P. jirovecii* in our cohort. The PcP is possible or not expected if the Ct is tested to be higher than 29.29. In total, 67 samples revealed a Ct higher than 29.29, of which for 62 patients, no PcP was suspected. Four patients were categorized to have a possible PcP (Ct: 30.2, 35.54, 40, 40) and one patient to have a probable PcP (Ct 30.52).

### 3.2. Characteristics of the Patients from Five Distinct Centers

The qPCR-positive samples (*n* = 109) consisted of 95 different patients (Table 1). Altogether, 70 non-HIV patients were categorized. A total of 3 patients were classified as proven, 43 as probable, and 13 as possible PcP, while 10 patients were not classifiable according to the revised EORTC/MSGERC criteria, and for 1 patient, no PcP was suspected. Regarding the HIV patients (*n* = 25), categorization revealed 2 proven, 6 probable, 16 possible, and 1 not categorizable PcP patients. The LAMP-positive samples (*n* = 70) consisted of 62 different patients, 21 HIV-positive and 41 non-HIV patients (13 transplant recipients, 11 solid tumor, 7 hematological malignancies, 6 other immunocompromising conditions and 4 autoimmune diseases). All samples were positive in the qPCR (Table 2).

### 3.3. Preanalytical Evaluation of the LAMP

For the positive control, the LAMP revealed a TTP of 14.00 min and 17.75 min in two samples, while the qPCR reached a Ct of 25.23 and 29.15, respectively. The negative controls showed no signal in both assays. The LAMP results for three samples for different storage conditions are listed in Table 3.

### 3.4. LAMP and qPCR Results

**Figure 2:**
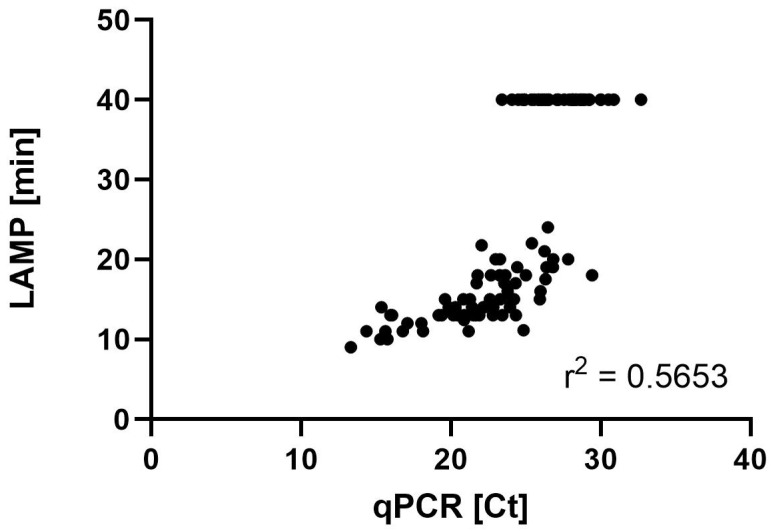
Correlation between qPCR and LAMP. Defining negative LAMP results in a TTP of 40 min in accordance with a negative Ct in qPCR; Pearson’s correlation r = 0.7519 (0.6561 to 0.8238; 95% confidence interval) revealed r^2^ = 0.5653.

Out of 109 analyzed samples, 6 (5.5%) were negative by qPCR and 38 (34.8%) by the LAMP. In qPCR, positive signals were detected for 103 (94.5%) samples. In contrast, the LAMP resulted in 70 (64.2%) samples with a positive signal, and 1 sample was invalid. Furthermore, 32 samples showed no signal in the LAMP, while qPCR was positive (Table 2). Out of these samples, 6 had a Ct lower than the threshold of 29.29 (Table 2). Consequently, 32 results were found to be false negative, while 1 false positive result was given by the LAMP. The LAMP’s specificity was 86%, the sensitivity was 68%, the PPV was 99%, and the NPV was 16% using Ct 29.29 as the cutoff for the comparison of both methods. Taking the clinical classification into account, all patients with proven PcP resulted in a positive LAMP. The LAMP was negative in 18 of 56 samples, categorized as probable PcP and in 14 of 36 samples classified as possible PcP. Correlation analysis of the samples, plotting Ct against TTP, showed an r^2^ of 0.5653, which indicated a low linear relationship between the TTP in the LAMP and the Ct obtained by the qPCR (Figure 2).

## 4. Discussion

The detection of *P. jirovecii* in respiratory samples is commonly performed via the microscopy of BALs and molecular methods, e.g., qPCR [5,10,11,12,13]. In the diagnostic pathway for the detection of PcP, first a microscopic examination of the respiratory samples is recommended by the guidelines, although this requires highly experienced personnel, irrespective of which staining method is used, which makes the diagnosis difficult to standardize [3,5,8]. The sensitivity of the microscopy methods varies between 26% and 44%, as found by the IFA, which is able to detect cysts and trophic forms [14,15]. For a more rapid and reliable detection of PcP, the LAMP has been established [16,17,18,19,20,21,22]. Since that time, different study groups have evaluated the LAMP [9,16,21,22,23,24]. In contrast to methods such as microscopy and qPCR, the LAMP is easy to handle and requires only a small amount of technical experience in molecular techniques [9,23,24]. At the same time, it offers an advantage to the qPCR regarding the time to the result, the practicability, and the robustness [23]. Here, a commercially available LAMP for PcP detection from respiratory material has been evaluated by testing a total of 109 samples from critically ill patients from five different university hospitals in Germany. The results were compared with a commercial qPCR assay, commonly used in routine diagnostics [23]. By evaluating the LAMP with statistical analysis, the PPV, NPV, sensitivity, and specificity can be calculated. The PPV was 99% and the NPV was 16%, the sensitivity was 68%, and the specificity was 86%, with Ct 29.29 used as a cutoff based on clinical features for the differentiation of colonization and infection with *P. jirovecii*. A Ct of 29.29 represents a fungal load of approximately 3 × 10^3^ copies per milliliter of sample. In our previous study, the PPV and NPV were 96% each, and the sensitivity and specificity were 84% and 99%, respectively, while we found a low correlation between the TTP of the LAMP and the fungal load of qPCR and the same a limit of detection (LOD) [9]. The LAMP eazyplex^®^ *Pneumocystis jirovecii* by AmplexDiagnostics evaluated here targets the *cox2* gene (GenBank accession no. MH010440.1), and in the current as well as previous study, we detected an LOD of 4 × 10^3^ copies/mL sample [9]. Regarding the correlation analysis, the LAMP assay showed a weak correlation between the TTP and the fungal load detected by the qPCR (Pearson correlation r = 0.7519 and r^2^ = 0.5653).

Huber et al. designed a German multicenter study, evaluating the eazyplex^®^ LAMP, including 49 patients with proven PcP and 126 patients without PcP [23]. For statistical analysis, ROC curve was carried out to evaluate the threshold of the qPCR to distinguish between a PcP and non-infected patients. They found that the sensitivity and specificity of the LAMP were 97.9% and 96.5%, respectively [23]. They set an LOD of 10^3^ copies of the *major surface glycoprotein* gene per 25 μL of BAL, corresponding to 10 to 20 *P. jirovecii* cells. This LOD was comparable to the LOD we defined in this study. Huber et al. also found a weak correlation between the TTP of the LAMP and the *β-tubulin* qPCR assay (Pearson correlation was r = 0.391) as well as the *mtLSU* rRNA qPCR (r = 0.353) [23]. Uemura et al. compared the performance of another LAMP with a real-time PCR-based method, reporting a diagnostic sensitivity of 87.5% with an LOD of 4 × 10^3^ copies/mL [16]. Furthermore, they described a decreasing TTP with a rising number of gene copies [16]. The overall diagnostic sensitivity of the LAMP assay using the initially established primer sets was high, and the LOD ranged between 50 and 100 copies/mL [17,18,19,20]. Since then, the LAMP for PcP detection has been modified by designing distinct primer sets. Wang et al. developed a LAMP with a new target site (mitochondrial 16s rRNA) in 2015 [21]. Subsequently, this LAMP assay resulted in an LOD of 10 to 50 copies/mL [21,22]. Varying LODs can probably be explained by different sample numbers and targets used for evaluation, while 10^3^ copies per milliliter of the considered gene seems to be the common LOD. In non-HIV patients, Perret et al. evaluated a cutoff of 5 × 10^3^ copies/mL to discriminate between a colonization and a PcP [25].

Huber et al. described the eazyplex^®^ LAMP as an appropriate tool to distinguish PcP infection from *P. jirovecii*-colonized patients [23]. Here, five of six samples turned out to be negative in the LAMP, considering samples with a Ct higher than 29.29. The one sample which was LAMP-positive showed a Ct of 29.44 (4.1 × 10^3^ copies/mL), just slightly above the cutoff value, and a TTP of 18 min. This patient had a solid tumor (prostate cancer), radiological signs for PcP in the lung, but no immunosuppressive therapy. No antifungal therapy was started after the diagnostic result was known. To investigate the power of the LAMP to distinguish between a colonization and an infection with *P. jirovecii*, a larger cohort of colonized patients is required. Because of changing fungal loads, depending on the underlying disease of the immunosuppression, there should be a big gray area in which no certain statement can be made. Analyzing the negative LAMP results, 39 (36%) samples were negative, although qPCR was positive. The Ct ranged between 23.41 and 32.7. In 34 samples, a Ct lower than 29.29 was observed. Altogether, 17 patients were categorized to have probable PcP, 14 to have possible PcP, and 7 were not classifiable. Nonetheless, clinical and radiological parameters have to be considered for reliably diagnosing PcP if the laboratory relies solely on the LAMP to detect *P. jirovecii*. Furthermore, β-1,3-D-glucan, as a fungal biomarker, should be included in the assessment [8]. Regarding only positive results, 69 positive samples from 62 patients were investigated, of whom 5 patients were categorized to have proven PcP, 34 probable, and 18 possible PcP, whereas 4 were not classifiable and 1 had no expected PcP. Regarding the patients’ clinical conditions together with a positive LAMP result, the basis for PcP therapy was given [16]. Taking the classification of patients as a foundation to interpret positive or negative results, all patients with proven PcP (*n* = 5; 100%) and 66% (37/56) of patients with probable PcP tested positive in the LAMP.

### Limitation

The limitations of our study are, among others, the storage conditions of the samples. Only the samples from Essen were analyzed both on arrival at the laboratory and after thawing. In this case, we showed the concordance of the qPCR and LAMP results. The very low NPV and the relatively low sensitivity observed might result from the analytical premises. Only samples from patients in whom PcP was investigated were included. Highly differing numbers of samples were sent from five different hospitals, so as a result, we observed a broadly distributed patient cohort, which might explain our findings. Samples from healthy individuals were not examined over the field. We defined a threshold based on clinical features to differentiate between infection and colonization due to *P. jirovecii* using an ROC analysis to set a cutoff in the qPCR. The results were not verified using microscopic examination. The variable nature of the specimen was not considered in detail and might result in unintended consequences. Samples from Erlangen could only be examined by qPCR in the Essen laboratory because only DNA already extracted from the original samples was sent to Essen.

## 5. Conclusions

The eazyplex^®^ LAMP is a useful method for the initial screening of patients suspected of PcP but not for excluding a diagnosis of PcP. The assay’s high detection limit means that most positive patients would not be colonized but would have true PcP.

## Figures and Tables

**Table 1:** Characteristics of patients.

Parameter	Results
No. of patients	95
Age (mean ± SD) (year)	59 ± 16.5
Sex (%)	
Male	62 (65.3)
Female	33 (34.7)
Underlying disease (no. of patients (%))	
HIV/AIDS	25 (26.3)
Solid tumor	20 (21.1)
Hematologic malignancy	20 (21.1)
Organ transplant	16 (16.8)
Kidney	11 (11.6)
Liver	3 (3.2)
Heart	1 (1.1)
Lung	1 (1.1)
Immunological disorder	7 (7.4)
Other	7 (7.4)

**Table 2:** Categorization of patient cohort and results in qPCR and LAMP.

Patient ID	Diagnosis	Categorization EORTC	qPCR (Ct)	Fungal Load (Copies/mL)	LAMP (TTP in min)
F-1	SO	probable	16.81	2.60 × 10^7^	11.87
F-2	SO	proven	18.04	1.00 × 10^7^	12.37
F-3	HIV	possible	20.89	1.10 × 10^6^	13.30
F-4	SO	probable	19.62	3.00 × 10^6^	15.32
F-5	HIV	proven	21.73	5.90 × 10^5^	17.68
F-6	AD	proven	22.23	4.00 × 10^5^	14.15
F-7	AD	probable	23.27	1.80 × 10^5^	20.75
F-8	ST	probable	22.98	2.20 × 10^5^	20.48
F-9	HM	probable	28.16	4.20 × 10^3^	negative
F-10	HIV	possible	27.08	9.50 × 10^3^	negative
F-11	SO	proven	26.39	1.60 × 10^4^	19.98
F-12	AD	possible	32.70	1.70 × 10^2^	negative
M-13	SO	probable	19.85	2.50 × 10^6^	14.85
M-14	ST	probable	21.90	5.20 × 10^5^	13.93
M-15	HIV	possible	19.18	4.20 × 10^6^	13.78
M-16	ST	probable	27.92	5.20 × 10^3^	negative
M-17	SO	probable	24.78	7.40 × 10^4^	negative
N-18	O	possible	23.31	2.00 × 10^5^	15.33
N-19	O	possible	21.79	6.30 × 10^5^	18.17
N-20	HIV	possible	19.39	3.80 × 10^6^	13.65
N-21	ST	probable	24.93	5.90 × 10^4^	negative
N-22	SO	probable	22.16	4.70 × 10^5^	14.05
N-23	HIV	possible	15.77	5.80 × 10^7^	10.47
F-24	HIV	proven	15.31	8.20 × 10^7^	10.32
F-25	HIV	possible	13.33	3.80 × 10^8^	9.90
ER-26	ST	probable	22.61	2.70 × 10^5^	15.75
ER-27	ST	probable	25.86	2.30 × 10^4^	negative
ER-27a			25.87	2.30 × 10^4^	negative
ER-28	HM	possible	24.51	6.40 × 10^4^	negative
ER-29	ST	probable	28.12	4.20 × 10^3^	negative
ER-30	ST	probable	25.57	2.90 × 10^4^	negative
ER-31	ST	probable	26.81	1.10 × 10^4^	19.75
ER-32	ST	probable	23.45	1.40 × 10^5^	13.75
ER-32a			23.95	9.80 × 10^4^	14.00
ER-32b			28.09	4.30 × 10^3^	negative
ER-32c			22.80	2.30 × 10^5^	13.75
ER-33	SO	probable	21.52	6.10 × 10^5^	13.00
ER-34	ST	not classifiable	30.88	5.00 × 10^2^	negative
ER-35	ST	not classifiable	22.68	2.60 × 10^5^	18.00
ER-36	HM	not classifiable	24.07	9.00 × 10^4^	negative
ER-37	HM	probable	23.78	1.10 × 10^5^	16.25
ER-38	O	probable	21.66	5.50 × 10^5^	13.78
ER-38a			27.56	6.50 × 10^3^	negative
ER-38b			24.92	4.70 × 10^4^	negative
ER-39	SO	not classifiable	29.22	1.80 × 10^3^	negative
ER-40	HM	possible	26.48	1.50 × 10^4^	24.75
ER-41	AD	not classifiable	28.85	2.50 × 10^3^	negative
ER-42	HM	possible	17.09	1.70 × 10^7^	12.67
ER-42a			23.26	1.90 × 10^5^	18.73
ER-42b			26.55	1.50 × 10^4^	negative
ER-43	AD	not classifiable	26.38	1.70 × 10^4^	negative
ER-44	O	probable	15.36	8.70 × 10^7^	14.52
ER-45	ST	not classifiable	23.63	1.40 × 10^5^	18.12
ER-46	HM	not classifiable	26.25	1.90 × 10^4^	26.00
ER-47	O	probable	30.00	1.00 × 10^3^	negative
ER-48	ST	probable	25.38	3.70 × 10^4^	negative
ER-48a			28.33	3.70 × 10^3^	negative
ER-49	HM	not classifiable	28.67	2.80 × 10^3^	negative
ER-50	AD	possible	15.63	7.10 × 10^7^	11.18
ER-50a			16.08	5.00 × 10^7^	13.20
ER-51	O	no PCP expected	24.35	8.10 × 10^4^	13.37
ER-52	HIV	not classifiable	14.36	1.90 × 10^8^	11.22
ER-53	ST	probable	26.25	1.90 × 10^4^	21.58
E-54	HIV	possible	28.14	3.10 × 10^3^	negative
E-55	SO	probable	23.57	1.10 × 10^5^	17.00
E-56	HIV	possible	27.14	6.90 × 10^3^	negative
E-57	HIV	possible	20.61	1.20 × 10^6^	13.00
E-57a			22.06	3.80 × 10^5^	522.00
E-58	HIV	possible	21.28	6.70 × 10^5^	15.00
E-59	SO	probable	24.86	4.40 × 10^4^	11.25
E-60	SO	probable	20.87	9.70 × 10^5^	12.75
E-61	SO	probable	21.41	6.20 × 10^5^	14.00
E-62	SO	probable	24.20	7.10 × 10^4^	15.00
E-63	O	possible	26.33	1.60 × 10^4^	17.00
E-64	HM	probable	28.63	2.40 × 10^3^	negative
E-65	SO	probable	24.44	6.60 × 10^4^	19.00
E-66	HM	probable	21.71	5.30 × 10^5^	13.00
E-67	HIV	probable	25.42	3.10 × 10^4^	22.25
E-68	HIV	probable	23.43	1.50 × 10^5^	13.25
E-69	ST	probable	25.98	2.00 × 10^4^	16.50
E-70	HIV	possible	15.97	1.17 × 10^8^	13.25
E-70a			23.41	1.40 × 10^5^	negative
E-71	HM	possible	28.39	6.50 × 10^3^	negative
E-72	HM	probable	27.16	1.70 × 10^4^	negative
E-73	HIV	possible	29.26	3.30 × 10^3^	negative
E-74	HIV	possible	20.29	3.90 × 10^6^	14.75
E-75	HIV	possible	23.82	3.40 × 10^5^	15.25
E-76	HIV	possible	25.95	6.30 × 10^4^	15.38
E-77	ST	probable	29.44	4.10 × 10^3^	18.03
E-77a			23.30	5.00 × 10^5^	15.70
E-78	HM	probable	26.06	6.60 × 10^4^	negative
E-79	HM	probable	21.20	7.50 × 10^5^	11.57
E-80	HM	no classifiable	27.84	4.80 × 10^3^	20.10
E-81	HM	probable	30.02	2.60 × 10^3^	negative
E-82	ST	possible	30.52	1.80 × 10^3^	negative
E-83	HIV	possible	20.16	3.90 × 10^6^	13.02
E-84	HIV	probable	25.00	8.80 × 10^4^	18.02
E-84a	HIV		18.14	1.90 × 10^7^	11.70
E-85	HIV	probable	20.84	2.30 × 10^6^	15.43
E-86	HIV	probable	20.46	4.70 × 10^6^	13.70
E-87	AD	probable	26.84	3.50 × 10^4^	20.97
E-88	ST	possible	28.76	1.70 × 10^3^	negative
E-89	SO	probable	25.24	2.70 × 10^4^	invalid
E-90	HM	probable	26.14	1.70 × 10^4^	negative
E-91	HM	probable	26.50	1.60 × 10^4^	negative
E-92	ST	probable	22.84	6.90 × 10^5^	14.78
E-93	HM	probable	28.94	2.30 × 10^3^	negative
E-94	HM	probable	24.32	8.30 × 10^4^	17.42
E-95	HIV	probable	16.78	2.60 × 10^7^	11.02

Abbreviations F: Freiburg; M: Munster; N: Nuremberg; ER: Erlangen; E: Essen; SO: solid tumor; HIV: Human Immunodeficiency Virus; AD: autoimmune disease; ST: solid organ transplantation; HM: hematological malignancy; O: other; Ct: cycle threshold; TTP: time to positivity; not classifiable due to lack of clinical data.

**Table 3:** LAMP results with different storage conditions.

Storage Conditions	Sample 1 TTP [min]	Sample 2 TTP [min]	Sample 3 TTP [min]
Initial	11:34	17:25	13:19
after two years of storage in −20 °C	14:04	16:37	15:29
24 h storage in 21 °C	14:53	20:06	13:39
48 h storage in 21 °C	14:54	20:16	12:19
72 h storage in 21 °C	14:04	18:19	12:56

## Data Availability

The original contributions presented in this study are included in the article. Further inquiries can be directed to the corresponding author.

## Abbreviations

The following abbreviations are used in this manuscript:

LAMP	loop-mediated isothermal amplification assay
*P. jirovecii*	*Pneumocystis jirovecii*
PcP	*Pneumocystis* pneumonia
NPV	negative predictive value
PPV	positive predictive value
r^2^	squared Pearson correlation coefficient
IFA	immunofluorescence assays
BAL	bronchoalveolar lavage
qPCR	quantitative real-time PCR
cox2	mitochondrial gene cytochrome c oxidase subunit 2
*mtLSU*	multicopy mitochondrial large-subunit rRNA (mtLSU) gene
EORTC/MSGERC	European Organization for Research and Treatment of Cancer and the Mycoses Study Group Education and Research Consortium
BS	bronchial secretions
Ct	cycle threshold
TTP	time to positivity
min	minutes
ROC	receiver operating characteristic
LOD	limit of detection
F	Freiburg
M	Munster
N	Nuremberg
ER	Erlangen
E	Essen
SO	solid tumor
HIV	Human Immunodeficiency Virus
AD	autoimmune disease
ST	solid organ transplantation
HM	hematological malignancy
O	Others
