# Correction: Scharmann et al. Evaluation of the Loop-Mediated Isothermal Amplification Assay (LAMP) Eazyplex^®^ *Pneumocystis jirovecii*. *J. Fungi* 2025, *11*, 300

**DOI:** 10.3390/jof11100752

**Published:** 2025-10-21

**Authors:** Ulrike Scharmann, Lisa Kirchhoff, Jan Buer, Franziska Schuler, Annerose Serr, Susann Rößler, Jürgen Held, Tobias Szumlanski, Joerg Steinmann, Peter-Michael Rath

**Affiliations:** 1 Institute of Medical Microbiology, University Hospital Essen, University of Duisburg-Essen, 45147 Essen, Germany; 2 Institute of Medical Microbiology, University Hospital Münster, 48149 Münster, Germany; 3 Institute of Medical Microbiology and Hygiene, Medical Center-University of Freiburg, Faculty of Medicine, University of Freiburg, 79104 Freiburg, Germany; annerose.serr@uniklinik-freiburg.de; 4 Institute of Medical Microbiology and Virology, University Hospital Carl Gustav Carus, TU Dresden, 01307 Dresden, Germany; 5 Institute of Microbiology—Clinical Microbiology, Immunology and Hygiene, University Hospital Erlangen, 91054 Erlangen, Germany; 6 Institute of Clinical Microbiology, Infectious Diseases and Infection Control, Paracelsus Medical University, Nuremberg General Hospital, 90419 Nuremberg, Germany

## Text Correction

In the original publication [1], we recognized a mistake in our manuscript which was published. In Section 2 Materials and Methods, we specified 125 µL buffer instead of 500 µL buffer for the preparation of the LAMP. However, this was a formal error. The test was carried out correctly, as the buffer provided by the company and ready for use was used for the preparation. This was not pipetted and the amount in milliliters was not changed. The main text in Section 2.2 should now read as follows:

“For the LAMP, 25 μL of sample was mixed up with 500 μL of buffer and incubated for 3 min at 99 °C and then added to lyophilized reagents.”

With the above corrections, the authors state that the scientific conclusions are unaffected. This correction was approved by the Academic Editor. The original publication has also been updated.
